# Bilateral Vertebral Artery Vasculitis—A Rare Manifestation of Giant Cell Arteritis and a Difficult Diagnosis Made Possible by 2-[^18^F]FDG PET/CT

**DOI:** 10.3390/diagnostics11050879

**Published:** 2021-05-14

**Authors:** Natasja Degn Justesen, Michael Stormly Hansen, Mads Radmer Jensen, Oliver Niels Klefter, Jane Maestri Brittain, Steffen Hamann

**Affiliations:** 1 Department of Ophthalmology, Rigshospitalet, University of Copenhagen, 2600 Glostrup, Denmark; michael.stormly.hansen@regionh.dk (M.S.H.); oliver.niels.klefter.01@regionh.dk (O.N.K.); steffen.ellitsgaard.hamann@regionh.dk (S.H.); 2 Faculty of Health and Medical Sciences, University of Copenhagen, 2200 Copenhagen, Denmark; 3 Department of Clinical Physiology and Nuclear Medicine, Bispebjerg & Frederiksberg Hospital, University of Copenhagen, 2400 Copenhagen, Denmark; mads.radmer.jensen@regionh.dk; 4 Department of Clinical Physiology, Nuclear Medicine and PET, Rigshospitalet, University of Copenhagen, 2100 Copenhagen, Denmark; jane.maestri.brittain@regionh.dk

**Keywords:** giant cell arteritis, large vessel vasculitis, 2-[^18^F]FDG PET/CT, temporal artery biopsy, bilateral vertebral artery vasculitis, vertebrobasilar insufficiency

## Abstract

Giant cell arteritis (GCA) is the most common form of large vessel vasculitis. GCA is a medical and ophthalmological emergency, and rapid diagnosis and treatment with high-dose corticosteroids is critical in order to reduce the risk of stroke and sudden irreversible loss of vision. GCA can be difficult to diagnose due to insidious and unspecific symptoms—especially if typical superficial extracranial arteries are not affected. In these cases, verification of clinical diagnosis using temporal artery biopsy is not possible. This example illustrates the diagnostic value of hybrid imaging with 2-deoxy-2-[^18^F]fluoro-D-glucose positron emission tomography/computed tomography (2-[^18^F]FDG PET/CT), and the limitations of the temporal artery biopsy in bilateral vertebral GCA, causing transient ischemic attack in the visual cortex. In addition it indicates that inflammation in the artery wall can be visualized on 2-[^18^F]FDG PET/CT despite long term and ongoing high dose glucocorticoid treatment.

**Figure 1:**
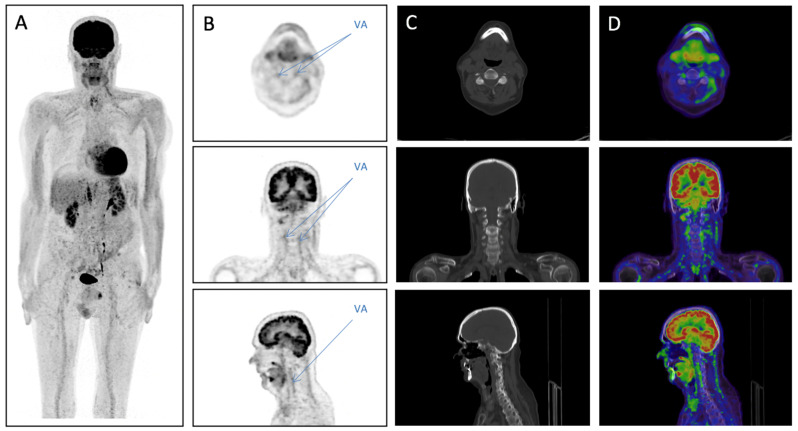
Whole body 2-[^18^F]FDG PET/low dose CT scan from vertex to the knees 32 days after start of corticosteroid treatment: (**A**) Maximum intensity projection showing normal 2-[^18^F]FDG uptake in the aorta and its large branches. Poor signal to noise ratio is likely due to ongoing high dose steroid treatment. Columns depict PET (**B**), lowdose CT, bone window (**C**) and fused PET/CT (**D**) in axial (upper row), coronal (middle row) and sagittal (lower row) reconstructions of the head and neck. Blue arrows show visible uptake in the vertebral artery interpreted compatible with giant cell arteritis. A 67-year-old male smoker with chronic obstructive pulmonary disease was referred to the Department of Internal Medicine due to unspecific symptoms including weight loss and pain in the neck and shoulders for three months. C-reactive protein was elevated at 47 mg/L (normal < 10 mg/L). A computed tomography (CT) scan of thorax and abdomen showed incidental adenomas in the adrenal glands bilaterally but no signs of malignancy or infection. A rheumatology consultation revealed additional history of unspecific bilateral visual disturbances as well as jaw claudication and scalp tenderness. Based on the initial clinical assessment, the rheumatologist suspected polymyalgia rheumatica (PMR) with a low probability of cranial involvement. Treatment with prednisolone 30 mg per day was initiated and a temporal artery biopsy (TAB) was requested along with an ophthalmological consultation. Four days later the ophthalmologist found no explanation to the visual symptoms and no signs of retinal or optic nerve ischemia. As the retinal and optic nerve assessment was unremarkable, the ophthalmologist suspected a retro chiasmal pathology of a transient/intermittent nature. In the setting of PMR the ophthalmology consult raised the suspicion of GCA and increased the dosage of prednisolone to 75 mg per day resulting in a decrease in CRP and marked symptom relief. The TAB result came back showing unspecific inflammation in the initial incisions of the temporal artery. In the more profound incisions of the artery a well-defined area with a transmural inflammation emerged involving adventitia media and intima. A combination of lymphocytes, neutrophils, eosinophils and epithelioid histiocytes were present. The internal elastic lamina was degraded. No convincing indication of giant cells were detected. The final conclusion from the pathologist was, that it was an arteritis with a suggestion of a granulomatous imprint, and although no giant cells were detected, GCA could not be ruled out. To pursue clinically suspected GCA a 2-[^18^F]FDG PET was performed 32 days after start of corticosteroid treatment (Figure 1). This showed faint but visible uptake in the vertebral arteries bilaterally which in the context was interpreted compatible with GCA. No Doppler ultrasound was performed. Hence, the reported visual symptoms can be explained by vertebrobasilar insufficiency causing ischemia in visual cortex, a rare manifestation of GCA. GCA is a chronic, idiopathic, granulomatous vasculitis of large- and medium-sized arteries with variable segmental distribution resulting in heterogeneous symptoms and diagnostic challenges. Incidence is higher in people of Northern European heritage and possible predispositions include genetic, environmental and autoimmune factors. GCA occurs exclusively in patients older than 50 years of age and is three times more frequent in women (24.2 per 100,000) than in men (8.2 per 100,000) [1]. Patients with cranial involvement (temporal arteritis) classically present with new-onset headache, fatigue, fever, muscle pain and jaw claudication and some suffer transient or permanent visual loss often due to ischemic optic neuropathy or central retinal artery occlusion. Therefore, GCA is a medical emergency indicating high-dose glucocorticoid treatment upon clinical suspicion. Patients with primary large artery involvement may present with unspecific symptoms such as malaise, fever, proximal muscle pain and weight loss and often pose a significant diagnostic challenge with possible differential diagnoses being other inflammatory diseases, e.g., polymyalgia rheumatica, infection and occult malignancy. Therefore, it is common practice to verify clinical suspicion using diagnostic tests. The gold standard for GCA diagnosis is by many considered to be TAB [2]. Even though TAB has a high specificity, the procedure suffers from low sensitivity and is a costly and invasive procedure [3]. Therefore, diagnostic imaging is recommended [4]. Ultrasound of extracranial arteries shows good diagnostic performance; however, one limitation is assessment of the vertebral artery, in which diagnostic criteria such as halo sign and compressibility cannot be assessed and flow disturbances are unspecific, as these may be caused by atherosclerosis. 2-[^18^F]FDG PET enables whole body visualization of artery inflammation but is limited by price and accessibility. In studies using 2-[^18^F]FDG PET as a first-line imaging modality for GCA the sensitivity was 92% and specificity was 85% compared to TAB [5]. In a study assessing 2-[^18^F]FDG PET for suspected large-vessel GCA, but with a negative biopsy, 2-[^18^F]FDG PET was found to be useful [6]. This study also found that corticosteroid therapy did not impact the diagnostic performance [6]; however, others have shown that corticosteroid therapy causes fast and significant reduction of 2-[^18^F]FDG uptake in extracranial arteries implying a narrow diagnostic window and high risk of false negative examinations if scanning is performed >10 days after treatment start [7]. This case illustrates that 2-[18F]FDG PET scans in patients with or without a positive TAB can be of diagnostic value if clinical suspicion of GCA is high.

## Data Availability

The data of this report are available from the corresponding author upon request.
